# Trocar sleeve adapter accelerates silicone oil injection in real-world vitreoretinal surgery

**DOI:** 10.1038/s41433-026-04279-6

**Published:** 2026-02-06

**Authors:** Philip Wakili, Colya N. Englisch, Clemens N. Rudolph, Charlotte Semoulin, Philipp K. Roberts, Clara E. Englisch, Marc A. Macek, Peter Szurman, Karl T. Boden

**Affiliations:** 1 Eye Clinic Sulzbach, Knappschaft Hospitals Saar, Sulzbach/Saar, Germany; 2 Department of Experimental Ophthalmology, Saarland University, Homburg/Saar, Germany; 3 Klaus Heimann Eye Research Institute (KHERI), Sulzbach/Saar, Germany

**Keywords:** Retinal diseases, Health care

## Abstract

**Purpose:**

This retrospective, non-randomised, observational cohort study investigated the efficiency of a novel 23G trocar adapter to accelerate silicone oil (SO) injection during endotamponading vitreoretinal surgery.

**Subjects:**

A total of 105 eyes were included consecutively. Only 23G procedures were eligible. Indications for SO injection were retinal detachment, recurrent retinal detachment, or SO exchange in eyes with retinal instability using either the high-flow viscous-fluid-extraction accessory cannula as the 23G trocar sleeve adapter (1362.VFE2, Dutch Ophthalmic Research Centre [DORC], Zuidland, the Netherlands; *n*_Densiron 68_ = 30, *n*_DORC Silicone 5000_ = 30) or the universal polyvinyl chloride (PVC) infusion tube as the standard method (1279.VFI, DORC; *n*_Densiron 68_ = 30, *n*_DORC Silicone 5000_ = 15). Eyes with aphakia, traumatic retinal conditions, or combined surgical procedures were excluded.

**Methods:**

Pars plana vitrectomy was performed, and after complete fluid–air exchange, SO was injected. The trocar sleeve adapter group was treated with an injection pressure of 2.5 bar and with the trocar valve removed. The standard group was treated with 4 bar pressure and with the valve left in place. The time needed to achieve functionally complete filling of the vitreous cavity with SO was recorded. Safety outcomes included the number of device disconnection events and the occurrence of any major complications.

**Results:**

Multiple linear regression analysis showed that the injection duration was significantly affected by SO type (*β* = 342.6, 95% confidence interval [CI]: 270.1–415.1, *p* < 0.0001), device type (*β* = 190.3, 95% CI: 117.2–263.5, *p* < 0.0001), and axial length (*β* = 40.3, 95% CI: 21.6–59.1, *p* < 0.0001). Application of the 23G trocar sleeve adapter significantly reduced the injection time needed to fill the vitreous cavity with Densiron 68, from 258.9 ± 74.7 s (PVC; 95% CI: 231.0–286.8; 241.5 ± 78 s [median ± interquartile range]) to 89.8 ± 55.0 s (95% CI: 69.3–110.4; 76.0 ± 50.5 s; *p* < 0.0001, Fisher’s LSD, two-way ANOVA). For DORC Silicone 5000, a time reduction from 773.7 ± 257.7 s (95% CI: 631.0–916.4; 835.0 ± 332.0) to 152.8 ± 44.5 s was achieved (95% CI: 136.2–169.4; 153.0 ± 66.5; *p* < 0.0001, Fisher’s LSD, two-way ANOVA). Comparisons of vitreous volume–adjusted injection durations calculated using the VIVEX formula yielded similar results (both *p* < 0.0001). Three disconnection events occurred, and all procedures were completed without major complications.

**Conclusions:**

Although the 23G trocar sleeve adapter was developed for SO extraction, it reduced the time required for low- and high-viscosity SO injection by three- and fivefold, respectively. The 23G trocar sleeve adapter thus has the potential to substantially shorten SO injection times in clinical practice, although randomised or crossover study designs will be required for validation.

## Introduction

Silicone oils (SOs) are widely used in vitreoretinal surgery as long-term endotamponades for complex cases. Low-viscosity SOs tend to cause vision-reducing emulsifications [1, 2], whereas high-viscosity SOs are limited by slow injection speeds [3, 4]. The time required to inject 6 mL of a high-viscosity SO is ~10 min [3, 4]. However, longer surgeries are associated with higher costs, increased infection risk, and enhanced light-induced retinal damage [5–7]. Moreover, with decreasing instrumentation size in vitreoretinal surgery, faster injection speeds—specifically for high-viscosity SOs, which remain critical for endotamponading—are necessary. Wilson et al. reported that the adoption of narrow-gauge cannulas has even prompted some surgeons to revert to low-viscosity SOs for easier injection, at the expense of a higher risk of early and extensive emulsification [8]. This trend underscores the importance of refining SO tamponade techniques to preserve therapeutic efficacy. Despite experimental evidence for trocar sleeve adapters [9], real-world clinical data remain limited, representing an important knowledge gap.

Viscosity, cannula length, and injection pressure all influence SO injection speed, but the inner diameter is the most critical factor, as it is its power of four, which is associated with injection speed. The mechanism is thus straightforward: sleeving the adapter over the trocar head instead of inserting a cannula within the trocar increases the inner diameter and thus the flow to the power of four at the tightest position of the SO injection system. While the trocar sleeve adapter investigated here is routinely used in SO extraction where it exhibits high performance [10], its implementation in SO injection has not yet been investigated in clinical practice, as aforementioned. This is because the negative pressure required for suction increases the adhesion between the trocar head and trocar sleeve adapter. Conversely, positive pressure, which is necessary for SO filling, can overload the device connection and promote disconnection of the trocar sleeve adapter from the trocar head. Therefore, the critical step is to titrate the injection pressure to ensure sufficient flow without risking adapter disconnection. We propose with this study a straightforward approach that permits faster SO filling while minimising this risk.

Our aim was thus to compare the injection time required for clinical application of the trocar sleeve adapter with that of a standard cannula technique using two commercially available SOs (*i.e*., one of low viscosity and the other of high viscosity). In other words, we investigated whether our promising laboratory results are applicable to real-world vitreoretinal surgery settings [9].

## Methods

### Study design

This study was designed as a retrospective, non-randomised, observational cohort study. All eyes were included consecutively. Case allocation followed a fixed alternating sequence independent of surgeon or indication. Five experienced vitreoretinal surgeons participated in the study, all using the same settings and the same vitrectomy platform for each approach, respectively. The trocar sleeve adapter having been part of routine clinical use for SO extraction for an extended period before the study, all surgeons were familiar with the handling of the device, and no relevant learning curve was expected. The adapter was not introduced at a specific time point, and its use did not chronologically shift.

Eyes were included if they underwent 23G vitrectomy with an indication for SO endotamponade, including retinal detachment, recurrent retinal detachment, or SO exchange in eyes with retinal instability. Exclusion criteria comprised aphakic eyes, eyes with traumatic retinal conditions, combined surgical procedures, and cases in which planned SO implantation was not performed intraoperatively. Written informed consent was obtained from all involved patients. The reported study adhered to the tenets of the Declaration of Helsinki and was approved by the local institutional review board (Ethikkommission bei der Ärztekammer des Saarlandes, 243/14).

### Endpoints

The time required to inject two different SOs using two different approaches was the main outcome measure. Corrected distance visual acuity (CDVA) and intraocular pressure (IOP) were measured preoperatively and postoperatively after 3 months and exploratorily reported.

### Injection tubes, cannulas, and trocars

The high-flow viscous fluid-extraction accessory cannula (1362.VFE2, DORC) was investigated as a 23G trocar sleeve adapter, and a universal polyvinyl chloride (PVC) infusion tube for 23G trocars was used as the control (1279.VFI, DORC). The valved 23G AVETA trocar systems (DORC) were used for each procedure. All devices were CE certified.

### Silicone oils

Densiron 68 (1400 mPa, Fluoron GmbH, Ulm, Germany) and Sil-5000-S SYRINGE (5000–5900 mPa, DORC) were used as low- and high-viscosity SOs, respectively.

### Surgery

**Fig. 1:**
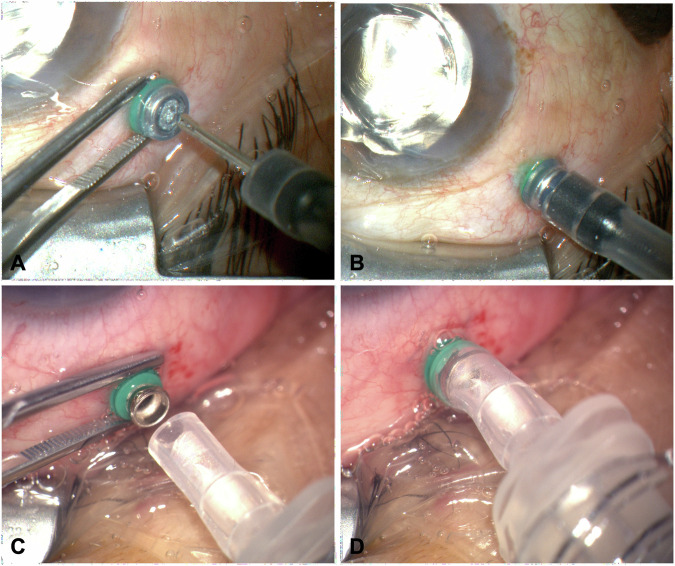
Application of the standard infusion tube and the 23G trocar sleeve adapter for silicone oil injection. Panels **A** and **B** show the standard infusion tube technique. Panels **C** and **D** show the placement of the 23G trocar sleeve adapter over the 23G trocar head after removal of the valve cap.

Pars plana vitrectomy was performed using a standard three-port 23G technique with a wide-angle visualisation system (RESIGHT, Carl Zeiss Meditec, Jena, Germany*)*. After complete fluid–air exchange and insertion of the flute needle, the vitreous cavity was filled with SO to a functionally complete level using either the standard infusion tube or the trocar sleeve adapter, as shown in Fig. 1. Complete filling was ensured by careful evacuation of residual air behind the intraocular lens under direct visualisation until the final intraocular air bubble was released. At this stage, a small column of SO typically ascended into the infusion line, which served as an additional indicator of functionally complete filling. Afterwards, the IOP was finely adjusted to 15–20 mmHg, measured using a regularly calibrated Schiötz tonometer, by SO addition or draining via the trocar. Instrument trocars were removed, and sclerotomies were closed with 8.0 Vicryl single-button sutures.

The light pipe was not routinely used to confirm filling and was applied only for videographic documentation in selected cases. The air infusion pressure was set at 25 mmHg and not reduced during SO injection. In our standard DORC EVA system configuration, the infusion pressure was ~30 mmHg, and the intraoperative IOP remained stable throughout the procedure.

#### Trocar sleeve adapter

For trocar sleeve adapter use, the valve cap of the valved 23G trocar was removed to allow placement of the adapter. Then, the SO tube of the EVA system was connected to the trocar using the trocar sleeve adapter. SO was injected intraocularly at a pressure of 2.5 bar using the same setup and protocol as described above.

#### Standard infusion tube

For standard infusion tube use, the valve remained in place within the valved 23G trocar system, where it was penetrated by the standard infusion cannula, which could then be inserted into the 23G trocar. SO was injected intraocularly at a pressure of 4 bar using the same setup and protocol as described above.

### Time measurement

The operating surgeon verbally initiated and terminated each measurement, and the elapsed time was recorded directly by the vitrectomy console. Timing started with the first foot pedal activation and ended with removal of the last air bubble, excluding fine IOP adjustment.

### Statistical analysis

Statistical analysis was performed using GraphPad Prism (v10.4.2). Continuous variables are reported as mean ± standard deviation (SD), median with interquartile range (IQR), and 95% confidence interval (CI). Normality was assessed using the Shapiro–Wilk test. Depending on the distribution, continuous variables were compared using Welch’s *t*-test or the Mann–Whitney *U* test; categorical variables were analysed using Fisher’s exact test.

A multiple least-squares linear regression was used to assess whether the injection duration was independently influenced by device type, SO type, age, or axial length. Subgroup analyses were performed twice: once using the raw injection duration and once after normalising the injection duration to ocular volume, calculated using the VIVEX formula based on axial length [11]. For group comparisons, a two-way analysis of variance (ANOVA) with Fisher’s uncorrected least significant difference (LSD) post hoc test was applied. ANOVA is generally robust to moderate deviations from normality; therefore, normality was not strictly required for all subgroups [12]. Secondary, exploratory outcomes, CDVA and the IOP time course, were also analysed using two-way ANOVA.

*p*-values were two-sided and considered statistically significant at *α* < 0.05. Post hoc power was calculated using G*Power 3 based on the ANOVA module (fixed effects, special effects, main effects, and interactions) and *α* = 0.05 [13].

## Results

### Demographics, baseline characteristics, CDVA, and IOP

A total of 105 eyes from 105 patients undergoing SO injection as part of endotamponading vitreoretinal surgery were analysed. Forty-five eyes were injected with SO via the standard PVC infusion line, and 60 eyes via the trocar sleeve adapter. In the standard-infusion-tube group, 15 eyes received DORC Silicone 5000 and 30 received Densiron 68. In the trocar-sleeve-adapter group, 30 eyes received DORC Silicone 5000 and 30 received Densiron 68.

Table 1 presents demographic and baseline characteristics of the study cohorts as well as CDVA and IOP time course. For exploratory CDVA analysis, two-way ANOVA was conducted to assess interaction as well as cohort and time effects; as the time factor was nonsignificant in one analysis, post hoc testing was restricted to between-group comparisons. Significant subgroup differences were observed for CDVA postoperatively in Densiron 68 eyes and preoperatively in Silicone 5000 eyes. For exploratory IOP analysis, all *F*-tests were nonsignificant, indicating stability across cohorts and over time (Table 1).

**Table 1:** Demographic and baseline characteristics, as well as the time course of corrected distance visual acuity (CDVA) and intraocular pressure (IOP).

	Densiron 68			Silicone 5000		
	Standard infusion tube	Trocar sleeve adapter	*p*	Standard infusion tube	Trocar sleeve adapter	*p*
Total (*n*)	30	30	—	15	30	—
Sex (*n*)						
Male	17	22	0.3^a^	10	14	0.3^a^
Female	13	8		5	16	
Age (Years)	62.0 ± 15.5	66.0 ± 14.3	0.3^b^	57.9 ± 20.1	62.8 ± 19.6	0.4^b^
Eye (*n*)						
Right	15	15	0.9^a^	10	19	0.9^a^
Left	15	15		5	11	
Axial length (mm)	24.2 ± 1.8	24.3 ± 1.2	0.8^b^	24.9 ± 3.9	23.6 ± 1.0	0.3^b^
Vitreous volume (cm^3^)	5.7 ± 1.4	5.8 ± 1.0	0.6^b^	6.8 ± 3.9	5.3 ± 0.8	0.9^c^
Lens status (phakic/pseudophakic)	5/25	4/26	0.9^a^	2/13	4/26	0.9^a^
Main diagnosis (*n*)						
Rhegmatogenous retinal detachment	26	23	0.5^a^	13	21	0.1^a^
Exudative retinal detachment	0	0		0	1	
Diabetic retinopathy	1	4		0	6	
Full-thickness macular hole	2	3		0	1	
Vitreous haemorrhage	1	0		0	1	
Macular pucker	0	0		1	0	
Bulbus hypotonia	0	0		1	0	
Surgery indication (*n*)						
Retinal detachment	16	16	0.9^a^	2	11	0.06^a^
Oil replacement	2	3		6	6	
Recurrent retinal detachment	9	9		5	13	
Other	3	2		2	0	
Corrected distance visual acuity (LogMAR)						
Preoperatively	1.4 ± 0.6	1.6 ± 0.6	0.2^e^	1.1 ± 0.7	1.5 ± 0.6	0.045^e^
Postoperatively	1.1 ± 0.5	1.5 ± 0.6	0.01^e^	1.2 ± 0.6	1.6 ± 0.5	0.1^e^
Intraocular pressure (mmHg)						
Preoperatively	15.7 ± 5.8	15.4 ± 8.0	ns^d^	14.2 ± 4.8	14.1 ± 5.5	ns^d^
Postoperatively	16.1 ± 6.8	14.5 ± 5.4		17.5 ± 7.0	14.0 ± 4.4	

Data are presented as counts or mean ± standard deviation.

^a^Fisher’s exact test.

^b^Welch’s *t*-test.

^c^Mann–Whitney *U* test.

^d^Two-way ANOVA.

^e^Fisher’s LSD test.

### Safety

No major complications occurred. The trocar sleeve adapter disconnected from the trocar in three cases (5%, 3 of 60 eyes): twice during Densiron 68 injection and once during DORC Silicone 5000 injection. In all instances, the adapter was reapplied without difficulty, and implantation continued uneventfully; the brief reconnection delays were included in the injection time measurements. No IOP spikes, sclerotomy leakage, SO misdirection, clinically relevant anterior chamber migration, or other safety-relevant complications were observed in either group.

### Silicone oil injection duration

Multiple linear regression showed that injection duration was significantly independently affected by SO type (*β* = 342.6, 95% CI: 270.1–415.1, *p* < 0.0001), device type (*β* = 190.3, 95% CI: 117.2–263.5, *p* < 0.0001), and axial length (*β* = 40.3, 95% CI: 21.6–59.1, *p* < 0.0001). Age had no significant effect (*β* = –0.1, 95% CI: –2.2 to 2.0, *p* = 0.9).

**Fig. 2:**
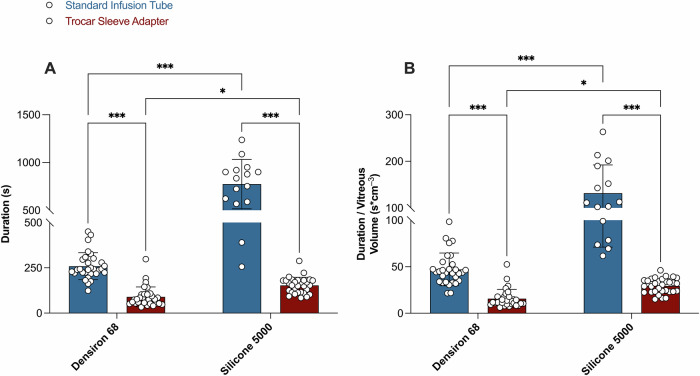
Silicone oil injection durations using the standard infusion tube and the 23G trocar sleeve adapter. Absolute **A** and vitreous volume–normalised **B** silicone oil injection duration. Bar graphs show mean ± SD for eyes subjected to the standard infusion tube (blue) or the trocar sleeve adapter (red), each supplied with Densiron 68 or DORC Silicone 5000. Each point represents an individual patient. * indicates *p* < 0.05, *** indicates *p* < 0.001.

Two-way ANOVA for subgroup analysis demonstrated a percentage of total variation of 31.3% for the SO factor (*p* < 0.0001), 58.5% for the device factor (*p* < 0.0001), and of 19.1% for interaction (*p* < 0.0001). In fact, the 23G trocar sleeve adapter significantly reduced the absolute injection time of Densiron 68 from 258.9 ± 74.7 s (95% CI: 231.0–286.8; median = 241.5 ± 78 s; *p* = 0.02, Shapiro–Wilk), obtained using the standard infusion tube, to 89.8 ± 55.0 s (95% CI: 69.3–110.4; median = 76.0 ± 50.5 s; *p* < 0.01, Shapiro–Wilk; *p* < 0.01, Fisher’s LSD; Fig. 2A), representing a threefold reduction. The trocar sleeve adapter also significantly reduced the injection time of DORC Silicone 5000 from 773.7 ± 257.7 s (95% CI: 631.0–916.4; median = 835.0 ± 332.0; *p* = 0.9, Shapiro–Wilk) to 152.8 ± 44.5 s (95% CI: 136.2–169.4; median = 153.0 ± 66.5; *p* = 0.1, Shapiro–Wilk; *p* < 0.0001, Fisher’s LSD; Fig. 2A), corresponding to a fivefold reduction. Supplement Video 1 illustrates the markedly accelerated SO injection achieved using the trocar sleeve adapter compared with that using the standard infusion tube.

Regardless of the application tool used, injection time was significantly shorter for the low-viscosity SO Densiron 68 than for the high-viscosity DORC Silicone 5000 (*p* = 0.03 and *p* < 0.0001, Fisher’s LSD, respectively).

For vitreous volume–normalised injection duration, the results were similar. The percentage of total variation was 27.4% (*p* < 0.0001) for the SO factor, 50.9% (*p* < 0.0001) for the device factor, and 14.3% (*p* < 0.0001) for interaction. The 23G trocar sleeve adapter significantly reduced the vitreous volume–normalised injection time of Densiron 68 from 47.1 ± 17.6 s cm⁻³ (95% CI: 40.6–53.7; median = 44.0 ± 19.4; *p* < 0.01, Shapiro–Wilk) using the standard infusion tube to 15.7 ± 9.9 s cm⁻³ (95% CI: 12.0–19.4; median = 12.4 ± 7.6; *p* < 0.01, Shapiro–Wilk; *p* < 0.0001, Fisher’s LSD; Fig. 2B). For DORC Silicone 5000, the trocar sleeve adapter reduced the normalised injection time from 131.6 ± 60.5 s cm⁻³ (95% CI: 98.1–165.1; median = 111.3 ± 111.4; *p* = 0.1, Shapiro–Wilk) to 29.3 ± 8.0 s cm⁻³ (95% CI: 26.3–32.3; median = 29.8 ± 12.5; *p* = 0.5, Shapiro–Wilk; *p* < 0.0001, Fisher’s LSD; Fig. 2B).

### Power analysis

Post hoc power analysis, performed on the absolute injection duration values, showed that with a partial *η*² of 0.75 and effect size of 1.73 for the device factor, the statistical power exceeded 0.95.

## Discussion

Instrument diameters are being increasingly reduced within vitreoretinal microsurgery. This exacerbates current issues such as long SO injection times during endotamponading surgery, specifically with respect to high-viscosity SOs.

The results of this retrospective, non-randomised, observational cohort study indicate that implementation of a 23G trocar sleeve adapter allows for a roughly threefold faster intraocular injection of Densiron 68 and roughly fivefold faster intraocular injection of DORC Silicone 5000 in comparison with injection using a standard infusion tube. These are highly important findings considering that faster SO injection means faster surgery, which in turn reduces the inhibition threshold of the surgeon to use SO, specifically highly viscous SOs, as well as the financial burden, postoperative infection risk, and light-induced retinal damage risk. Given that every minute in the surgery room is expensive, and that the 23G trocar sleeve adapter is currently at a similar cost level as the standard infusion tube, use of the adapter promises to reduce the financial burden, as previously discussed [9].

Although, to the author's knowledge, there is no evidence yet, indicating that surgery duration of endotamponading vitrectomy is a risk factor for postoperative endophthalmitis [14, 15], failing to decrease vitreoretinal surgery duration entails unnecessarily extended risk exposure. Reducing light-induced retinal damage is one of the surgeon’s priorities to improve visual outcome [16, 17]. For instance, intraoperative light filters were developed to weaken retinal phototoxicity [17]. Nevertheless, surgery time remains critical in reducing light-induced retinal damage via reducing the duration of retinal light exposure [18]. Thus, the three- and fivefold time reductions demonstrated herein also provide a protective layer for patients’ visual outcome. This is even more important because other parts of the surgery are difficult to accelerate, making SO injection optimisation one of the keys to reducing the surgery time. While the lack of CDVA time course in the cohorts does not support the concept of SO-injection-duration-dependent light-induced retinal damage, this observation (including IOP analysis) should be interpreted with caution; the analyses were exploratory and underpowered for this heterogeneous outcome, being influenced by multiple confounding factors. In addition, recent literature has highlighted that a small subset of patients may develop unexplained visual loss during tamponade or after SO removal, despite the absence of identifiable anatomical or functional causes [19]. While this rare phenomenon was not the focus of our study, its growing recognition underlines the need for continued vigilance and further research into SO-related safety considerations.

Although the importance of high-viscosity SO remains undiminished, only a few studies have investigated the issue of injection duration. Of course, SOs differ with respect to injectability, as previously investigated [3]. In addition to device-associated improvements, the surgeon can reduce time by increasing the sclerotomy and ultimately use larger device lumens. However, this not only undermines the principle and strenghts of minimally invasive surgery, but also carries the disadvantage of losing decisive amounts of time with suturing. In terms of device-associated improvements, in 2022, Hammer *et al*. [4] assessed polyimide-lined cannulas for SO injection within an experimental setup. They demonstrated a time decrease of 70%–77% for injecting various SOs (Siluron 1000, Siluron 2000, Siluron 5000, Siluron Xtra, Densiron 68, and Densiron Xtra) in contrast to the injection time required using a standard metal cannula for 23G trocars. However, to the author's knowledge, until now, no follow-up study has been published investigating the potential of polyimide-lined cannulas for SO injection in clinical practice. Moreover, the wasted potential of using the full inner diameter would remain.

In a similar laboratory context, our trocar sleeve adapter demonstrated even greater performance in reducing SO injection duration for five different SOs (Siluron 1000, Siluron 2000, Siluron Xtra, Densiron 68, and DORC Silicone 5000), achieving a time savings of up to 90% while applying a 4 bar injection pressure (vs. 6 bar used by Hammer et al. [4] [9]). However, for clinical application, trocar sleeve adapter requires an injection pressure of no more than 2.5 bar to avoid disconnection, as the adapter sits loosely on the trocar head. In this study, disconnection was observed only in three cases, whereby reconnection was simple and successful in all cases. In the end, the practical reasons for disconnection are that the trocar sleeve adapter was not optimally placed over the trocar or the surgeon had not generated a suitable haptic counterpressure onto the eye. Overall, the approach is efficient and reliable, although technical upgrades, such as installing a screw or clip between the trocar and trocar sleeve adapter, might enhance the reliability of the connection and even allow for higher injection pressures,and eventually, greater time savings.

One important question arises: Why did we achieve a tenfold reduction in an experimental setting for five SOs ranging from low-to-high viscosity, while only obtaining a threefold reduction for low-viscosity SOs and fivefold reduction for high-viscosity SOs in real-life vitreoretinal surgery? The first reason for this discrepancy is the ocular counterpressure, which slows the flow towards the intravitreal space. Second, in our laboratory study, we used an injection pressure of 4 bar for both devices [9], whereas in the clinical setting, the trocar sleeve adapter was applied at an injection pressure of 2.5 bar; the standard infusion tube was operated using 4 bar. Therefore, the observed time reduction reflects the performance of the complete trocar sleeve adapter configuration, including its specific pressure requirement and valve handling, rather than the effect of the adapter interface alone.

In conclusion, our findings demonstrate the clinical potential of the 23G trocar sleeve adapter to substantially accelerate low- and high-viscosity SO injection. Disconnections are rare when the recommended injection pressure of no more than 2.5 bar is used. Nevertheless, the surgeon must maintain a suitable haptic counterpressure on the eye while ensuring precise vertical alignment with the trocar to facilitate smooth SO implantation and minimise the risk of disconnection. Future improvements include the implementation of an appropriate screw or clip mechanism with which a secure connection can be achieved, further increasing the injection pressure and potentially accelerating SO injection. While the present data indicate substantial time savings under routine conditions, confirmation in prospective randomised or crossover studies will be required to confirm the effect. The investigated adapter system has been approved in more than 30 countries, making it a good option to easily and substantially accelerate SO-based endotamponading vitreoretinal surgery worldwide.

## Summary

### What was known before

- The injection of silicone oil (SO) endotamponades is a critical time factor in vitreoretinal surgery. Low-viscosity SOs can be injected relatively quickly but tend to emulsify, while high-viscosity SOs resist emulsification but are very slow to inject.

### What this study adds

- Implementation of a 23G trocar sleeve adapter, originally designed for oil extraction, significantly accelerates SO injection. The device reduces injection times by approximately 3-fold for a low-viscosity SO (Densiron 68) and 5-fold for a high-viscosity SO (DORC Silicone 5000), indicating a substantial potential to shorten overall surgical duration.

## Supplementary information

Video 1

## Data Availability

The data that support the findings of this study are available from the corresponding authors upon reasonable request.
